# Malignant Melanoma in Children and Adolescents Treated in Pediatric Oncology Centers: An Australian and New Zealand Children’s Oncology Group (ANZCHOG) Study

**DOI:** 10.3389/fonc.2021.660172

**Published:** 2021-04-29

**Authors:** Anne L. Ryan, Charlotte Burns, Aditya K. Gupta, Ruvishani Samarasekera, David S. Ziegler, Maria L. Kirby, Frank Alvaro, Peter Downie, Stephen J. Laughton, Siobhan Cross, Timothy Hassall, Geoff B. McCowage, Jordan R. Hansford, Rishi S. Kotecha, Nicholas G. Gottardo

**Affiliations:** ^1^ Department of Haematology, Oncology and Bone Marrow Transplant, Perth Children’s Hospital, Perth, WA, Australia; ^2^ Children’s Cancer Centre, The Royal Children’s Hospital, Melbourne, VIC, Australia; ^3^ Cancer Centre for Children, The Children’s Hospital at Westmead, Westmead, NSW, Australia; ^4^ Kids Cancer Centre, Sydney Children’s Hospital, Randwick, NSW, Australia; ^5^ Department of Haematology/Oncology, Women’s and Children’s Hospital, Adelaide, SA, Australia; ^6^ Department of Haematology/Oncology, John Hunter Children’s Hospital, Newcastle, NSW, Australia; ^7^ Department of Haematology/Oncology, Monash Children’s Hospital, Melbourne, VIC, Australia; ^8^ Starship Blood and Cancer Centre, Starship Children’s Hospital, Auckland, New Zealand; ^9^ Children’s Haematology/Oncology Centre, Christchurch Hospital, Christchurch, New Zealand; ^10^ Department of Haematology/Oncology, Queensland Children’s Hospital, Brisbane, QLD, Australia; ^11^ Murdoch Children’s Research Institute; Department of Pediatrics, University of Melbourne, Melbourne, VIC, Australia; ^12^ Telethon Kids Cancer Centre, Telethon Kids Institute, Perth, WA, Australia; ^13^ Curtin Medical School, Curtin University, Perth, WA, Australia

**Keywords:** cutaneous melanoma, childhood, dermatology, outcome, rare tumors

## Abstract

**Objectives:**

Unlike adults, malignant melanoma in children and adolescents is rare. In adult melanoma, significant progress in understanding tumor biology and new treatments, including targeted therapies and immunotherapy have markedly improved overall survival. In sharp contrast, there is a paucity of data on the biology and clinical behavior of pediatric melanoma. We report a national case series of all pediatric and adolescent malignant melanoma presenting to ANZCHOG Childhood Cancer Centers in Australia and New Zealand.

**Methods:**

A retrospective, descriptive, multi-center study was undertaken to identify patients less than 18 years of age treated for cutaneous malignant melanoma over a twenty-year period (1994 to 2014). Data on clinical characteristics, histopathology, and extent of disease, treatment and follow-up are described.

**Results:**

A total of 37 cases of malignant melanoma were identified from all of the Australasian tertiary Childhood Cancer Centers. The median age was 10 years (range 1 month – 17 years). Clinically, the most common type of lesion was pigmented, occurring in sixteen (57%) patients, whilst amelanotic was seen in 7 patients (25%). In 11 (27.9%) the Breslow thickness was greater than 4mm. A total of 11 (29.7%) patients relapsed and 90% of these died of disease. Five-year event free survival (EFS) and overall survival were 63.2 (95% CI: 40.6 – 79.1) and 67.7% (95% CI: 45.1 – 82.6) respectively.

**Conclusion:**

Our data confirms that melanoma is a rare presentation of cancer to tertiary Australasian Childhood Cancer Centers with only 37 cases identified over two decades. Notably, melanoma managed in Childhood Cancer Centers is frequently at an advanced stage, with a high percentage of patients relapsing and the majority of these patients who relapsed died of disease. This study confirms previous clinical and prognostic information to support the early multidisciplinary management in Childhood Cancer Centers, in conjunction with expert adult melanoma centers, of this rare and challenging patient group.

## Introduction

Cutaneous melanoma in children and adolescents is rare, with an incidence ranging between 0.3 and 1 per 100 000 children a year, and only a small percentage occurring before puberty (1–4). Pediatric melanoma has not been studied as extensively as adult melanoma and our current understanding of the outcomes for melanoma presenting in children and adolescents is limited to mainly single-institution review series and a recent prospective European rare pediatric cancer consortium registry study (3–7). In Australia <2% of all cases of cutaneous melanoma occur before the age of 25 years (1, 7–9). Given that certain geographical areas of Australia and New Zealand have been reported to have the highest rates of adult melanoma in the world, it is important to review pediatric data and evaluate specific factors that influence prognosis and overall survival (7–9).

The rarity of pediatric melanoma combined with differences in the clinical presentation compared to adults (10), especially in young children can make diagnosis challenging. Moreover, histopathological diagnosis is complicated due to the similar histological appearance of malignant melanoma with more benign lesions in childhood, such as spitz nevi, atypical spitz nevi and the concept of melanocytic tumors of uncertain prognosis (MELTUMP). Molecular diagnostic tools, such as fluorescent *in situ* hybridization and genomic testing, are now assisting pathologists to distinguish between these different entities (11, 12).

Over the past decade, significant advances have been made in elucidating the molecular pathogenesis of adult melanoma. Approximately 60% of adult melanoma patients have identifiable oncogenic mutations in the *BRAF* gene, whilst another 20% have oncogenic *NRAS* mutations (13). These genetic discoveries have been translated into the clinic, with mitogen-activated protein kinase (MAPK) pathway inhibitors such as BRAF and mitogen-activated protein kinase/extracellular signal-regulated kinase (MEK) inhibitors inducing dramatic responses and significantly improving survival (14, 15). In addition, the highly immunogenic nature of melanoma has successfully been exploited using immunotherapy, with major improvements in patient outcomes (16).

Despite these advances, melanoma that has spread to distant sites remains incurable in the majority of patients. Clinical trials are ongoing to develop novel and more effective targeted therapies and immunotherapies to treat metastatic melanoma (16–19). The management of pediatric melanoma patients has been extrapolated from the treatment of adults with melanoma. However, the limited understanding surrounding the diagnosis and prognosis of childhood melanoma initially led to the almost uniform exclusion of these patients from clinical trials offered to adult patients; a strategy that has hampered research efforts and access to treatment in this population (20). The increased use of precision medicine to molecularly characterize tumors in children has further guided specific treatments including molecular target therapies and immunotherapy. In Australia this is being undertaken through the Precision Medicine in Children with Cancer (PRISM) clinical trial (https://clinicaltrials.gov/ct2/show/NCT03336931).

For these reasons, we evaluated the clinical characteristics and outcomes of all pediatric and adolescent malignant melanoma patients managed at pediatric oncology centers in Australia and New Zealand over the past two decades.

## Methods

We undertook a retrospective, descriptive, multicenter study of children and adolescents with malignant melanoma. All ten pediatric oncology centers in the Australia and New Zealand Children’s Oncology Group (ANZCHOG) participated in the study. Patients aged less than 18 years and treated for malignant melanoma between 1994 and 2014 were included. A detailed review of each patient chart was undertaken and data collected for each case included age, gender, ethnicity, site of disease, staging, extent of disease (including Breslow thickness), ulceration and *BRAF* status. Treatment outcomes, mode of follow-up, relapse and cause of death were also recorded. All data were all collected in accordance with the approval of institutional research ethics boards.

The number of cases of pediatric and adolescent melanoma patients was compared to the number of cases published in the national cancer registry, for both Australia and New Zealand, over the same timeframe.

Data has been presented as medians and ranges and as percentages. The overall survival (OS) has been calculated according to the Kaplan-Meier method: from the date of diagnosis to the date of death or latest follow-up for patients still alive. The event free survival (EFS) has been calculated from the date of diagnosis to the date of disease recurrence, death or latest follow-up for patients still alive and in complete remission.

## Results

Thirty-seven patients with malignant melanoma were identified over the twenty-year period timeframe. The ratio of males to females was 1:1 and the median age at diagnosis was 10 years age (range 1 month to 17 years). A total of 16 (43%) patients were less than 10 years old. The majority of patients were of Caucasian ethnicity (83.7%) with only five New Zealand Maori (n=3), African (n=1) and Australian Aboriginal (n=1) patients. Tumors were located on the head and neck (n = 14, 37.8%), trunk (n = 10, 27%), upper limb (n = 5, 13.5%) and lower limb (n = 5 cases, 13.5%). The primary location was unknown in two patients (**Table 1**).

**Table 1:** Patient characteristics and Clinical Features of the 37 patients with malignant melanoma in Australia and New Zealand 1^st^ January 1995 – 31^st^ December 2014.

	N (%)
Gender: Male/Female	18/19 (48.6/51.4)
Age:	
0 – 4 years	5 (13.6)
5 – 9 years	11 (29.7)
10 – 14 years	13 (35.1)
15 – 18 years	8 (21.6)
Ethnicity:	
Caucasian	31 (83.7)
African	1 (2.7)
Aboriginal	1 (2.7)
Maori	3 (8.1)
Unknown	1 (2.7)
Site of Disease:	
Trunk	10 (27)
Head and Neck	14 (37.8)
Extremity - Upper	5 (13.5)
Extremity - Lower	5 (13.5)
Other	1 (2.7)
Unknown	2 (5.6)
Histology:	
Superficial Spreading	4 (10.8)
Nodular	8 (21.6)
On congenital naevus	6 (16.3)
Spitzoid	8 (21.6)
Not classified	11 (29.7)
Breslow Thickness:	
≤ 1.00mm	4 (10.8)
1.01 – 2.00mm	4 (10.8)
2.01 – 4.00mm	6 (16.3)
> 4.00mm	11 (29.7)
Unknown	12 (32.4)
AJCC Stage at diagnosis:	
Stage I	8 (21.6)
Stage II	9 (24.3)
Stage III	4 (10.8)
Stage IV	11 (29.7)
Unknown	5 (13.6)

The Australia and New Zealand cancer registries reported 1,778 children and adolescents with melanoma over the same twenty-year time period (**Table 2**).

**Table 2:** Incidence Count from 1^st^ Jan 1994 – 31^st^ Dec 2013, based on Australia and New Zealand national cancer registry data.

0 – 4 years	19
5 – 9 years	43
10 – 14 years	198
15 – 19 years	1289
Number of Deaths: Australia 1st Jan 1994 – 31st Dec 2013	
0 – 4 years	1
5 – 9 years	2
10 – 14 years	3
15 – 19 years	26
Incident Count: New Zealand 1st Jan 1994 – 31st December 2013	
0 – 4 years	3
5 – 9 years	3
10 – 14 years	30
15 – 19 years	193
Number of Deaths: New Zealand 1st Jan 2007 – 31st December 2012	
0 – 4 years	1
5 – 9 years	0
10 – 14 years	1
15 – 19 years	8

Mean population of children 0 - 19 years in Australia and New Zealand over the duration of the study = 3, 069, 745 per annum (http://www.stats.govt.nz/topics/population, https://abs.gov.au/statistics/people/population.

Melanoma arose from congenital nevi in six patients (16.3%) and two patients had a history of malignancy with one patient being treated for acute leukemia, including total body radiation conditioning for an allogeneic bone marrow transplant and another patient with previous anaplastic astrocytoma and leukemia and known Li Fraumeni Syndrome.

A description of lesions at clinical presentation was available in 28 patients. The majority (16 cases, 55%) had a pigmented lesion reported, whilst seven (25%) had amelanotic lesions which were described as scaly, warty or friable in appearance. Two (7%) patients presented with subungal nodular lesions on the toe and index finger and three (11%) patients had nodal enlargement as the presenting clinical feature.

Histologically the most common melanoma subtypes were nodular and Spitzoid, with eight cases (21.6%) reported for each group respectively. Breslow thickness was reported in 25 cases and nearly 30% (11 cases) had thick lesions with a measurement greater than 4mm at presentation.

Based on the American Joint Committee on Cancer (AJCC) classification, our study found that eight patients (21.6%) were stage I, nine patients (24.3%) were stage II, four patients were stage III (10.8%) and the remaining were stage IV (11 cases, 29.7%) at diagnosis. For five patients, no exact staging classification was possible. *BRAFV600E* testing was conducted in seven (18%) patients and was positive for one patient. There was also one patient who was tested for and found positive for an *NRAS* exon 3 mutations.

### Initial Treatment

All but three patients underwent initial surgical resection of their tumor. Of the three patients who did not receive surgical resection, one had an unknown primary lesion; one initially had a shave biopsy before proceeding to further surgery and one had a fine needle aspirate of an enlarged lymph node before subsequent nodal excision. Primary re-excision, in order to obtain adequate margins, was performed in seven (19%) of patients. Lymph node biopsy was undertaken in 13 (35%) patients and lymphoscintigraphy was performed in 1 patient. Lymph nodes were positive following biopsy in five (38%) patients. Among the cases with positive lymph node biopsies, three had nodular histology and two were associated with congenital nevi. All but one of these patients relapsed and subsequently died of the disease.

Chemotherapy was used in five patients, following initial surgical resection, and included interferon in four patients (3 patients stage III and 1 stage IV) and a combination of cisplatin, dacarbazine and fotemustine in another patient (stage IV).

### Relapses and Treatment

Eleven patients (29.7%) relapsed, with a median time from diagnosis to first relapse of 22 months (range 2 months – 9 years). All but one of the 11 patients who relapsed died from malignant melanoma. Among the patients who relapsed, 3 (27%) had melanoma arising from a congenital nevus, four (37%) had nodular histology, one (9%) had superficial spreading histology and in three (27%) patients the histology was unknown. The site of relapse was in regional lymph nodes for five patients, local cutaneous for two patients and metastatic in four patients. A summary of relapsed treatment can be found in **Table 3**. Relapsed treatments were varied and included surgery, when feasible (five cases), but more predominately chemotherapy (seven cases) and palliative radiotherapy (five cases). Targeted therapy was used in two patients and included immunotherapy with ipilimumab and pembrolizomab in one patient and the combination of targeted therapy with the *BRAF* inhibitor dabrafinab followed by ipilimumab in the other patient.

**Table 3:** Relapse characteristics.

**Site of Primary Disease**	**Site of Relapse**	**Age at Diagnosis**	**Time to relapse**	**Histology**	**Breslow Thickness**	**AJCC Stage at Diagnosis**	**Therapy for Relapse**	**Outcome**
Subungal	Metastatic (Scalp, lymph nodes)	7 years, 3 months	21 months	Nodular	3.5mm	II	Chemotherapy Radiotherapy	DOD
Trunk	Trunk	20 months	2 months	On congenital naevus	8mm	IV	Chemotherapy Radiotherapy	DOD
Ear	Metastatic (lymph nodes, Lung)	11 years, 7 months	18 months	Nodular	10mm	IV	Surgery Chemotherapy Immunotherapy	DOD
Trunk	Trunk	5 years, 7 months	10 months	On congenital naevus	7.4mm	IV	Surgery Radiotherapy	DOD
Scalp	Metastatic (lymph nodes, bone, liver)	15 years, 4 months	8 months	Superficial Spreading	3.2mm	IV	Surgery, Chemotherapy, Immunotherapy Radiotherapy	DOD
Lymph node	Lymph nodes	17 years, 3 months	2 years, 4 months	Unknown	Unknown	IV	Chemotherapy	DOD
Scalp	Lymph nodes	13 years, 6 months	1 year, 2 months	Nodular	7.5mm	II	Chemotherapy	DOD
Trunk	Lymph nodes	16 years, 2 months	9 months	Nodular	6.4mm	II	Chemotherapy Immunotherapy	DOD
Meninges	Lymph nodes	3 years, 9 months	1 year, 5 months	Unknown	Unknown	Unknown	Surgery	DOD
Trunk	Metastatic (lymph nodes, liver, bone)	1 year, 6 months	9 months	On congenital naevus	11.4mm	II	Radiotherapy	DOD

### Survival Outcomes

**Figure 1:**
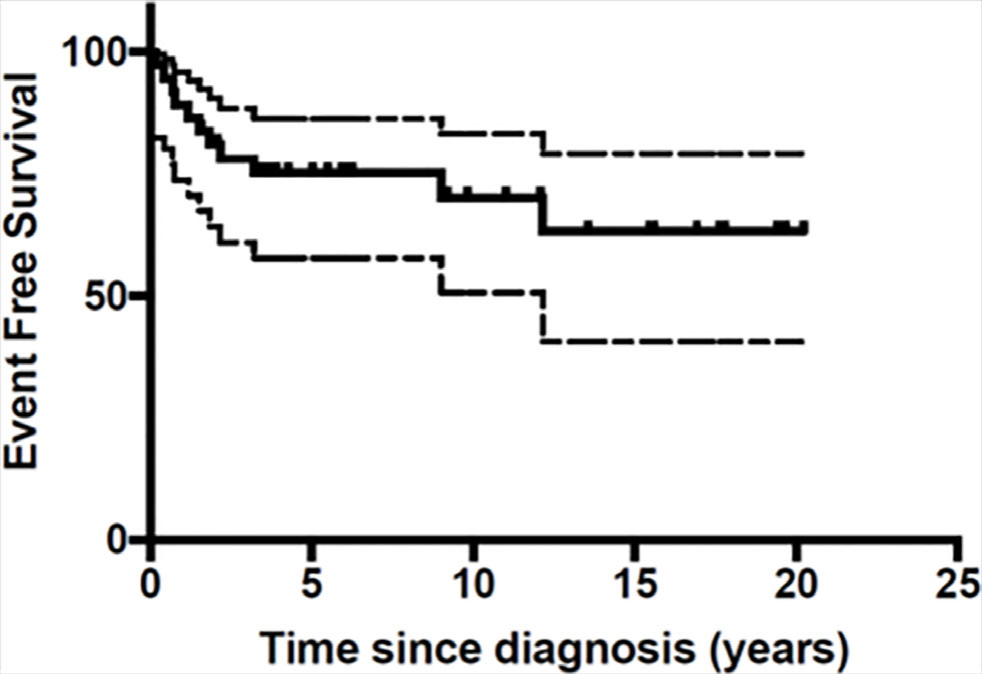
Event Free Survival (EFS) of the 37 patients with malignant melanoma.

**Figure 2:**
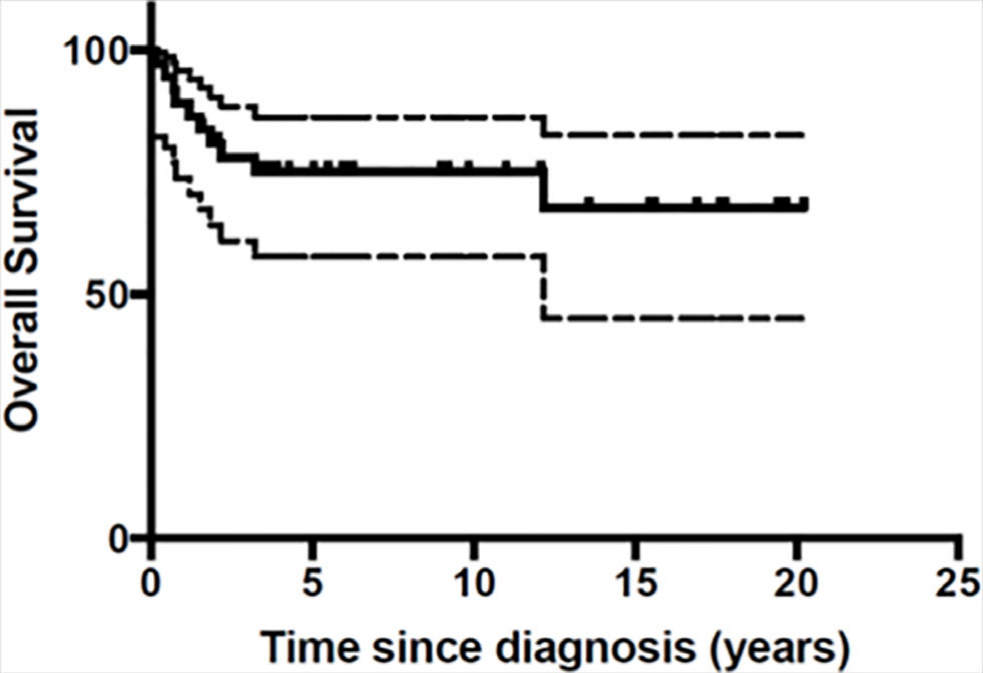
Overall Survival (OS) of the 37 patients with malignant melanoma.

At the end of the follow-up period 10 patients (27%) had died of disease (**Table 3**). A total of 26 patients were still in first Complete Response (CR) and one in second CR. **Figures 1** and **2** show Kaplan-Meier curves for EFS and OS. After a median follow-up of 5.8 years (2 months – 16.5 years) the 5 year EFS and OS were 63.2 (95% CI: 40.6 – 79.1) and 67.7% (95% CI: 45.1 – 82.6) respectively.

## Discussion

We report here a retrospective case series of all malignant melanomas occurring in children and adolescents in Australia and New Zealand from the ANZCHOG, who were managed at tertiary pediatric oncology centers over two decades. Patients were referred from various other health professionals, including dermatologists, primary health care physicians general and plastic surgeons, often after initial surgery.

Our finding that only 37 pediatric patients were treated at tertiary pediatric oncology centers over a twenty-year period is in stark contrast with national registry data, where the total incidence of malignant melanoma among children and adolescents in Australia and New Zealand over the same time period was 1,778 across both registries. These data reveal that malignant melanoma in children and adolescents is rarely treated at pediatric oncology centers in Australia and New Zealand. In keeping with our data, a recent Italian study estimated that only one in three children and one in ten adolescents with melanoma are treated in tertiary pediatric referral centers (3). However, there is a paucity of similar data for other countries.

Another important aspect of our findings concerns the stage of the melanoma in referred patients. Our data shows that a large percentage (29.7%) of patients referred to tertiary oncology centers had stage IV disease at presentation. Our findings strongly suggest that patients are usually referred to tertiary oncology centers only when harboring advanced stages of the disease, which likely also explain the relatively high death rate of 27% that was observed. Consistent with our hypothesis, Réguerre et al., analyzed 52 cases of malignant melanoma in children and adolescents and suggested that the relatively poor prognosis noted in their cohort could be explained by having selected patients referred to expert oncology hospitals (21). Compared to other published series, patients reported by Réguerre et al. had more advanced stages or the worst initial presentations, such as metastatic relapse after the excision of a supposedly benign lesion.

For a cancer like melanoma, early diagnosis is a crucial factor determining the outcome of a patient. For both adults and children, the successful management of melanoma is stage dependent and surgical treatment alone, with adequate margins, is curative for both adult and pediatric patients who present with early-stage localized disease (22, 23). In addition, early diagnosis has also been shown to significantly improve the quality of life for patients with melanoma. For example, a study of 395 melanoma patients evaluated with the EQ-5D-5L questionnaire showed that postoperative stage I–II melanoma patients experienced better health outcomes >2 years after treatment, compared to patients with stage III melanoma (24, 25).

The morphological appearance of lesions is also of paramount significance. The presence of thick lesions (>4mm) is associated with a higher risk of disease spread, and these patients may benefit from additional chemotherapy (26). In a large pediatric series of melanoma, Brecht et al., reported that the presence of histological ulceration, nodular histology, Breslow thickness of more than 2mm and AJCC classification of III or IV, were indicative of a poor prognosis (27). Our data, although contained a relatively small number of cases, is consistent with these findings, with tumor thickness, nodular histology and advanced stage having worst survival outcomes (All six patients with known nodular histology and Breslow thickness of >2mm died of disease). However, these histological factors need to be evaluated further in larger cohorts of children and adolescents with melanoma before their true prognostic value can be evaluated. The presence of ulceration was rarely documented and its prognostic significance should be stressed in the histopathological work-up of such cases in the future. Lymph node evaluation with the use of sentinel node biopsy was not routinely documented in this cohort but would offer additional important prognostic information for childhood melanoma patients. In addition, other missing information relating to comorbidities, family history and further details of treatment such as surgical techniques and margins would have been valuable to evaluate in relation to survival outcomes. Another important aspect concerns treatment location. It is difficult to ascertain whether the location of diagnosis and treatment for children and adolescents with malignant melanoma ultimately influences outcome. It is well recognized that children and adolescents diagnosed with cancer benefit from access to a specialized multidisciplinary team with ongoing systematic clinical reviews and surveillance imaging (21, 28, 29). A recent Italian study that analyzed nationwide hospital discharge of adolescents with melanoma found that patients were dispersed across a large number of hospitals, not always in a pediatric oncology center. The study identified 418 adolescents diagnosed with cutaneous melanoma between 2007 and 2014. These patients were referred to 137 different hospitals, where they were treated in various units, such as pediatric and adult oncology, adult general surgery and dermatology. These findings highlight the need to develop better ways to manage melanoma patients, to ensure that they are referred to an appropriate specialized clinic (28).

Given the association between childhood and adolescent malignant melanoma and the presence of an underlying cancer predisposition syndrome, any such case should be considered for referral to clinical genetics and for genetic counseling (30–32).

As in adults, changes in the appearance of a pigmented lesion should alert to the possibility of melanoma. However, the ABCDE clinical rule (asymmetry, border, irregularity, color variability and diameter >6mm and evolving), often used to identify concerning skin lesions in adults, may be difficult to apply to children (33). Common benign lesions such as Spitz nevi and benign nevi that grow as the child grows often have these clinical features. A study by Cordoro et al. showed that 60% of children aged 0 to10 years and 40% of children aged 11 to 19 years with melanoma did not present with the conventional ABCDE criteria, but rather with amelanosis, bleeding, uniform color and *de novo* development were the most common clinical presentations (33). In our cohort, while the majority of patients had pigmented lesions, a large number were described as amelanotic and associated with non-specific skin changes or bleeding. The low index of clinical suspicion for malignant melanoma in such lesions has been reported as the cause of delays or misdiagnosis in 50 to 60% of patients (21, 26).

Despite the advances in targeted therapies of adult melanoma, the genomic landscape of pediatric melanoma has only recently been explored. Only 18% of our cohort underwent analysis of *BRAF* V600E mutation, which was present in only one case. In addition, a patient in which melanoma arose from a congenital nevus was positive for an *NRAS* mutation. The limited molecular information in this study reflects the era over which many of the patients were treated; molecular analyses, especially for tumors such as melanoma, were still in their infancy and not widely available. Such molecular information is now essential and should be collected in future prospective clinical studies to fully characterize this rare childhood malignancy and to potentially guide treatment with targeted therapies a. Indeed, a study by Lu et al. provides the most comprehensive genomic analysis of pediatric melanoma to date (34) and shows that there are three distinct groups of childhood melanoma, each with a unique clinical behavior and molecular profile. The first group is the conventional melanoma that shares the histopathological and clinical features of adult melanoma, where 50 – 60% of patients harbor the *BRAF* V600E mutation and the condition rarely develops before puberty. The second group arises in association with congenital nevi, where approximately 5 – 10% of all patients with large or giant congenital nevi develop melanoma. The condition arises most often in the first decade of life and harbors *NRAS* mutations. Finally, the third group is Spitzoid melanoma, where *NRAS* and *BRAF* mutations are absent and the lesions often have a less aggressive clinical course (34).

Collectively, the data from these initial genomic studies suggest that the therapeutic targets for genotype specific melanoma in adults might be applicable to some cases of melanoma in children. What remains to be determined is the safety and efficacy of targeted therapies currently used in adults in children and adolescents with malignant melanoma. Consequently, it is critical that the molecular pathogenesis of future cohorts of pediatric melanoma lesions be evaluated to continue to resolve these important clinical issues.

Due to the rarity of malignant melanoma in young people, it has been difficult to conduct prospective clinical trials tailored to children. In addition, most adult treatment protocols are generally not accessible to children. Recent approval and early phase trials with immune checkpoint inhibitors, such as ipilimumab and nivolumab, *BRAF* inhibitors (e.g. Dabrafanib) and MEK inhibitors (e.g. Binimetinib) has begun for adolescents with advanced malignant melanoma at selected pediatric centers (35). In this study, only 2 out of the 11 children with relapsed disease were treated with immunotherapy. This was due to them being treated in an era prior to immunotherapy being an established treatment for metastatic melanoma and not due to contraindications to the use of immunotherapy.

## Conclusion

Whilst the limited number of cases identified in this study precludes any definitive conclusions on the clinical behavior of melanoma in children and adolescents, some important observations can be made. Consistent with previous reports, the diagnosis of malignant melanoma is challenging, especially in young children as their clinical and histopathological features are poorly characterized. The cases we identified have been compared to published national cancer registry data and build on previous international studies revealing that only a small proportion of children and adolescents with malignant melanoma are managed in tertiary oncology centers (3, 21, 28). Malignant melanoma patients treated in these centers often have more advanced disease and subsequent poor prognosis.

As with many rare pediatric cancers, the diagnosis and subsequent treatment of malignant melanoma is challenging. This study confirms previous clinical and prognostic information in pediatric melanoma to support the early multidisciplinary management in Childhood Cancer Centers, in conjunction with expert adult melanoma centers, of this rare and challenging patient group (21, 25, 28). Scientific advancement together with growing collaborative efforts provide opportunities to advance understanding and treatment (34–36). Further progress involves taking advantage of sophisticated molecular analysis and application of this knowledge in the clinical setting, such that a therapeutic multi-center prospective trial, which includes the collection of tumor samples, be considered in the near future.

## Data Availability Statement

The raw data supporting the conclusions of this article will be made available by the authors, without undue reservation.

## Ethics Statement

The studies involving human participants were reviewed and approved by Child and Adolescent Health Service- Western Australia GEKO - Governance Evidence Knowledge Outcomes. Written informed consent from the participants’ legal guardian/next of kin was not required to participate in this study in accordance with the national legislation and the institutional requirements.

## Author Contributions

AR: conception design, data acquisition, analysis and interpretation, and manuscript drafting. CB: data acquisition. AG: data acquisition. DZ: conception designs and data acquisition. MK: data acquisition and manuscript drafting. FA: data acquisition. PD: data acquisition. SL: data acquisition. SC: data acquisition, and analysis and interpretation. TH: data acquisition, and analysis and interpretation. GM: data acquisition, and analysis and interpretation. JH: conception design, data acquisition, analysis and interpretation, and manuscript drafting. RSK: conception design and manuscript drafting. NG: conception design, data acquisition, analysis and interpretation, and manuscript drafting. All authors contributed to the article and approved the submitted version.

## Conflict of Interest

The authors declare that the research was conducted in the absence of any commercial or financial relationships that could be construed as a potential conflict of interest.
